# Risk-Reducing Breast and Gynecological Surgery for BRCA Mutation Carriers: A Narrative Review

**DOI:** 10.3390/jcm12041422

**Published:** 2023-02-10

**Authors:** Serena Bertozzi, Ambrogio P. Londero, Anjeza Xholli, Guglielmo Azioni, Roberta Di Vora, Michele Paudice, Ines Bucimazza, Carla Cedolini, Angelo Cagnacci

**Affiliations:** 1 Breast Unit, University Hospital of Udine, 33100 Udine, UD, Italy; 2 Ennergi Research (Non-Profit Organisation), 33050 Lestizza, UD, Italy; 3 Department of Neuroscience, Rehabilitation, Ophthalmology, Genetics, Maternal and Infant Health, University of Genoa, 16132 Genova, GE, Italy; 4 Academic Unit of Obstetrics and Gynecology, IRCCS Ospedale San Martino, 16132 Genoa, GE, Italy; 5 Anatomic Pathology Unit, Department of Surgical Sciences, and Integrated Diagnostics (DISC), University of Genoa, 16132 Genoa, GE, Italy; 6 Anatomic Pathology Unit, IRCCS Ospedale San Martino, 16132 Genoa, GE, Italy; 7 Department of Surgery, Nelson R. Mandela School of Medicine, University of KwaZulu Natal, Durban 4001, South Africa

**Keywords:** risk-reducing surgery, pathogenetic gene mutations, BRCA, gene mutation carrier, breast cancer, ovarian cancer, endometrial cancer

## Abstract

This narrative review aims to clarify the role of breast and gynecological risk-reduction surgery in BRCA mutation carriers. We examine the indications, contraindications, complications, technical aspects, timing, economic impact, ethical issues, and prognostic benefits of the most common prophylactic surgical options from the perspectives of a breast surgeon and a gynecologist. A comprehensive literature review was conducted using the PubMed/Medline, Scopus, and EMBASE databases. The databases were explored from their inceptions to August 2022. Three independent reviewers screened the items and selected those most relevant to this review’s scope. BRCA1/2 mutation carriers are significantly more likely to develop breast, ovarian, and serous endometrial cancer. Because of the Angelina effect, there has been a significant increase in bilateral risk-reducing mastectomy (BRRM) since 2013. BRRM and risk-reducing salpingo-oophorectomy (RRSO) significantly reduce the risk of developing breast and ovarian cancer. RRSO has significant side effects, including an impact on fertility and early menopause (i.e., vasomotor symptoms, cardiovascular disease, osteoporosis, cognitive impairment, and sexual dysfunction). Hormonal therapy can help with these symptoms. Because of the lower risk of developing breast cancer in the residual mammary gland tissue after BRRM, estrogen-only treatments have an advantage over an estrogen/progesterone combined treatment. Risk-reducing hysterectomy allows for estrogen-only treatments and lowers the risk of endometrial cancer. Although prophylactic surgery reduces the cancer risk, it has disadvantages associated with early menopause. A multidisciplinary team must carefully inform the woman who chooses this path of the broad spectrum of implications, from cancer risk reduction to hormonal therapies.

## 1. Introduction

The expanding understanding and management of breast cancer risk factors denote an essential step in preventing this malignancy, just like any other neoplastic illness. There are some heritable, non-modifiable risk factors that the patient carries with her at a genetic level, in addition to the patient’s age, gender, and modifiable factors (e.g., tobacco smoke, alcohol consumption, or diet).

In particular, it is estimated that about 5% of breast cancers have a hereditary basis [1], with a prevalence of about 0.85–3.0% of germline pathogenic BRCA variations among breast cancer patients (20–60% of all hereditary breast cancers) [2,3,4,5]. Meanwhile, about 20% of ovarian cancers are thought to be hereditary, and almost 32% are supposed to be caused by pathogenic mutations in the BRCA1 and BRCA2 genes [6,7]. In many cases, this number is lower due to the fact that not all patients are tested for gene mutations, especially patients with a high probability of being positive, to contain the costs of this type of strategy. 

In addition to the best-known pair of genes that can predispose a patient to the development of breast cancer, namely the BRCA1 and BRCA2 genes, there are other genes with variable penetrance that can in some way increase the predisposition to this type of neoplasm. In patients with negative genetic tests for BRCA mutations, panels that include these genes are often used to rule out other types of inheritance. BRCA1 gene mutations cause familial breast-ovarian cancer-1 (BROVCA1), and BRCA2 gene mutations cause familial breast-ovarian cancer-2 (BROVCA2). Up until the age of 80, women who are BROVCA1-sensitive have a cumulative risk of 72% for breast cancer and 44% for ovarian cancer [8,9,10]. However, BROVCA2-sensitive women have a cumulative risk of 17% for ovarian cancer and 69% for breast cancer at the same age [8,10]. 

**Figure 1:**
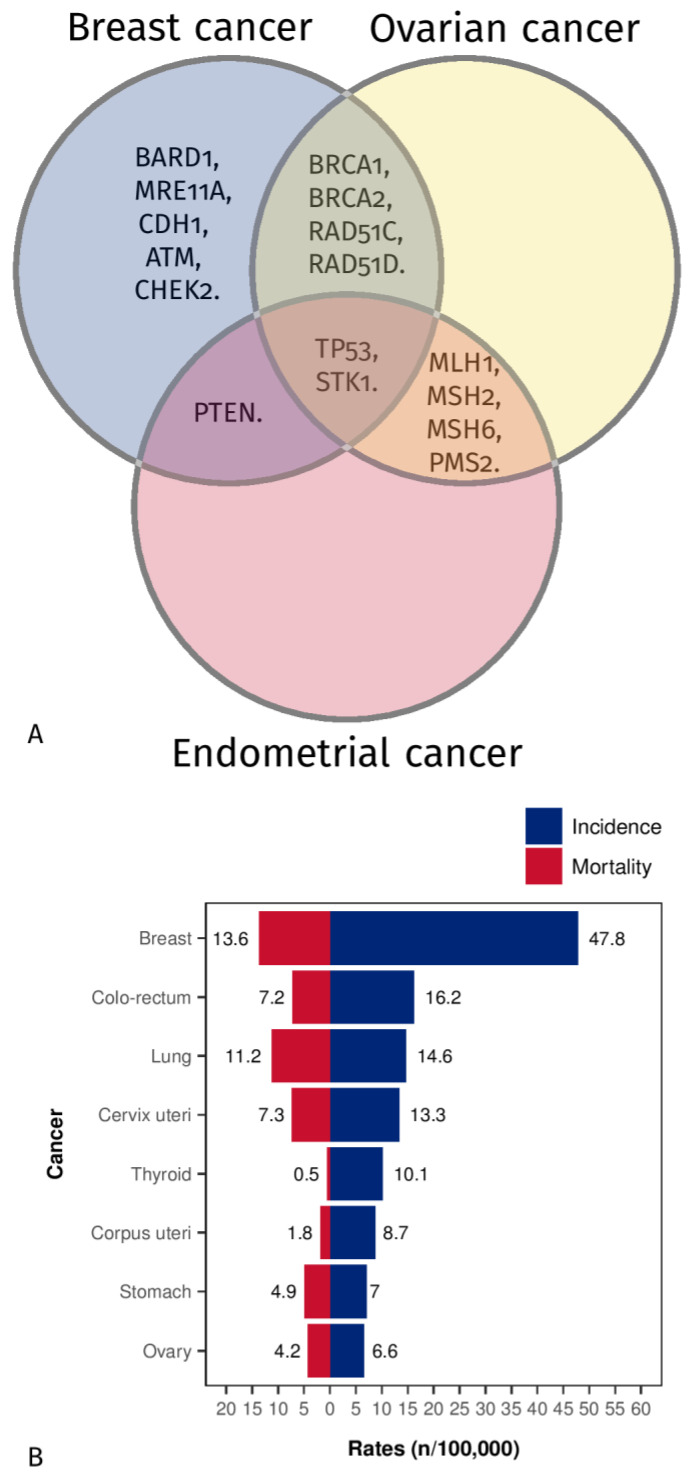
(**A**) Venn diagram showing mutated predisposing genes shared among hereditary ovarian, breast, and endometrial cancer [7]. (**B**) Estimated global age-standardized tumor incidences and mortality rates (female population only) according to GLOBOCAN data in 2020 (www.iarc.fr—World Health Organisation (WHO), access on 22 November 2022).

**Figure 2:**
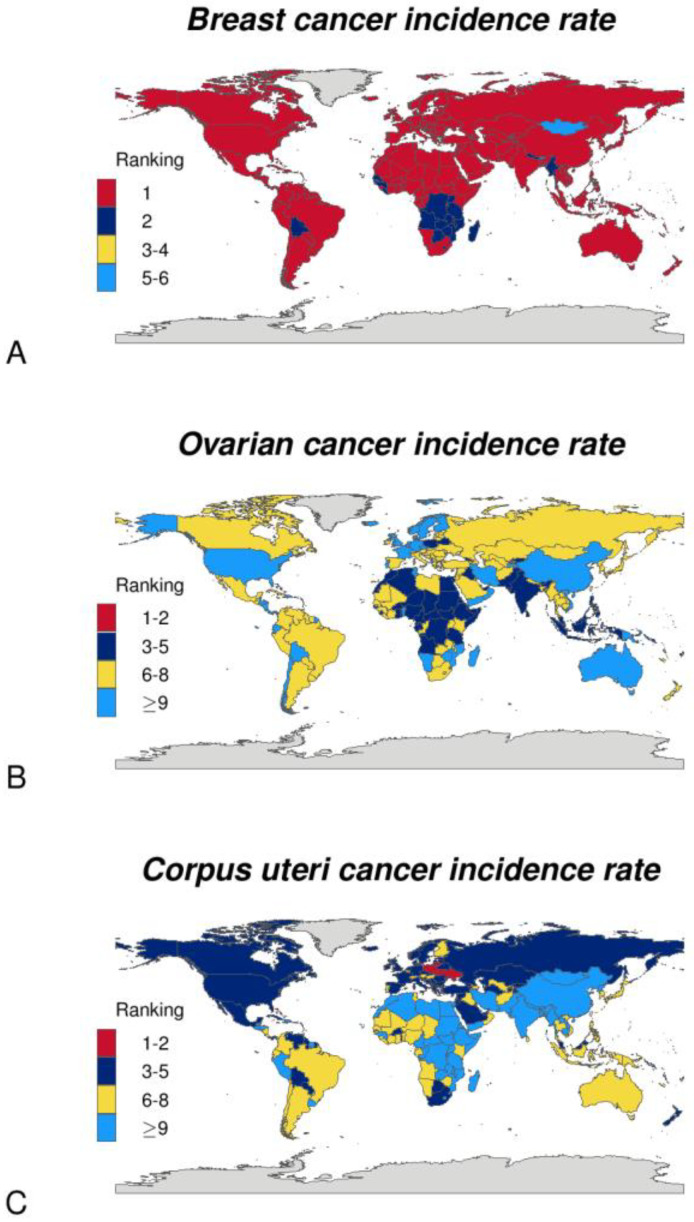
Rankings of estimated age-standardized incidence rates (World) in female population in 2020. (**A**) Breast cancer. (**B**) Ovarian cancer. (**C**) Corpus uteri cancer.

Hereditary ovarian and breast cancer share various mutated predisposing genes (Figure 1A) [7]. Some causative mutated genes are also shared with endometrial cancer (Figure 1A) [7]. These three cancers are among women’s top ten most frequent cancers (Figure 1B). According to GLOBOCAN data, the 2020 global estimated incidence of breast cancer was 47.8 for every 100,000 women, and it was the most frequent cancer in most countries worldwide (Figure 1B and Figure 2A) (www.iarc.fr, accessed on 22 November 2022). Endometrial cancer was the sixth most frequent cancer, and ovarian cancer was the eighth (estimated global average data, Figure 1B and Figure 2B,C). Meanwhile, the mortality rates showed breast cancer to be the most common, followed in order by ovarian and endometrial cancer (estimated global average data, Figure 1B) (www.iarc.fr, accessed on 22 November 2022). 

Undoubtedly, the knowledge of predisposing gene mutations currently offers, in addition to some interesting new therapeutic options, such as the use of PARP inhibitors for patients with mutations of the BRCA genes, various prophylactic options of surgical and non-surgical types. This narrative review aims to clarify the role of breast and gynecological risk-reducing surgery in patients with BRCA mutations. In detail, we discuss the indications, contraindications, complications, technical aspects, timing, economic impact, ethical issues, and prognostic benefits of the most common prophylactic surgical options currently offered to patients carrying BRCA mutations, which we observe from the points of view of a breast surgeon and a gynecologist.

## 2. Materials and Methods

A comprehensive literature assessment was performed by querying the following databases: PubMed/Medline, Scopus, and EMBASE. The databases were systematically searched from their inception to 6 August 2022. The queries’ specifics are displayed in Table 1. Three authors independently screened the items and extracted the more pertinent ones within the scope of this review. On the whole, 5638 items were discovered, leaving 4385 after duplicates were eliminated. After the titles and abstracts were manually screened, 50 items were deemed pertinent for this review, and their entire texts were evaluated. In addition, 90 records were identified from manual searching, the screening of selected item references, and expert consultation. The relevance and scientific merit of the publications chosen for this evaluation were considered. The assessment of a scientific paper’s worth was based on its full-text publication in a peer-reviewed journal, ignoring works that were retracted later. Relevance was based on the following tenets: pragmatism, which included the most valuable articles to provide a thorough overview, beginning with literature reviews; pluralism, which had as many perspectives as possible; and contestation, which discussed competing data and dissenting arguments.

## 3. Findings

### 3.1. Prophylactic Breast Surgery

Over the years, several techniques for bilateral risk-reducing mastectomy (BRRM) have been used, including risk-reducing skin-sparing mastectomy and risk-reducing nipple-sparing mastectomy (RRNSM). Nipple-sparing mastectomy was linked to improved psychosocial and sexual well-being and a higher quality of life [11,12,13].

RRNSM consists of completely removing the mammary gland through a minimal scar, saving the skin and the nipple–areola complex. Access to this procedure varies based on the preferences and personal experience of the breast surgeon. The most frequently used incisions are radial or “italic S” within the upper-outer quadrant, along the inframammary sulcus, or periareolar with a possible radial extension. For the dissection of the gland from the subcutaneous and prefascial layers, various devices can be used to promote hemostasis and reduce perioperative complications (i.e., a LigaSure sealing system or an Ultracision device).

This procedure can be bilateral in the case of healthy mutation carriers or unilateral in the case of patients submitted to contralateral breast cancer surgery. In the latter case, risk-reducing surgery can be offered at the same time as the contralateral oncological intervention, or it can be deferred if the surgical risks should be minimized to not compromise the optimal timing of any possible indicated adjuvant therapy.

Reconstruction in these cases can be offered immediately during the same operative session, except in patients at high risk of complications, for which the plastic surgeon deems it more appropriate to perform a two-stage reconstruction (i.e., heavy smokers, uncontrolled diabetes, previous chest irradiation, and patients with tissues damaged for any other reason). Postoperative complication occurrence, based on recent studies, is reported to be around 43%, and thus not very far from the postoperative complication rate of breast reconstruction after oncological surgery independent of prophylactic indications, and additional surgery for medical or aesthetic purposes was required in about 72% of cases [14,15,16]. However, the current literature shows very promising results related to the long-term aesthetic outcomes of BRRM [17].

Considering that the cumulative risk of breast cancer by age 80 is estimated to be 72% and 69% for BRCA1 and BRCA2 mutation carriers, respectively [8], the largest recent study about prophylactic mastectomy confirmed it to be an effective risk-reducing strategy for BRCA gene mutations, leaving an extremely low risk of new ipsilateral breast cancer development [18]. In particular, BRRM is ascertained to reduce the risk of breast cancer by 90% in patients with the BRCA mutation [19].

**Figure 3:**
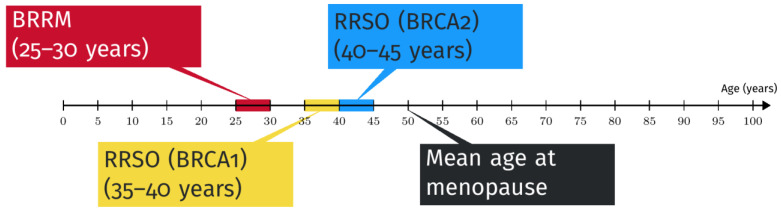
Timing of risk-reducing procedures [20].

Taking into account the mean age of the development of breast cancer in the case of BRCA mutation, the optimal age to undergo prophylactic breast surgery is between 25 and 30 years, as described in Figure 3 [20].

The prevalence of occult breast tumors in prophylactic mastectomy surgical specimens in some series exceeds 11%, despite negative preoperative radiological findings [16,21], and is more likely with a personal history of breast cancer, age over 60, and Breast Imaging Reporting and Data System (BI-RADS) category 4 findings (suspicious for malignancy) on preoperative imaging [22]. However, there is usually no indication for the intraoperative evaluation of the retroareolar tissue, and the sentinel lymph node biopsy can be safely omitted in the case of BI-RADS category 1–3 findings (negative, benign, or probably benign) on preoperative breast magnetic resonance imaging (MRI) [22]. 

Focusing on BRCA mutation carriers already diagnosed with breast cancer, although a meta-analysis demonstrated a contralateral breast cancer risk reduction in patients undergoing contralateral risk-reducing mastectomy (CRRM) for high familial or genetic risk (RR 0.04; 95% CI 0.02–0.08) or an ascertained BRCA mutation (reduced by 91–93%; RR 0.07; 95% CI 0.04–0.15) [23,24,25], there is still great debate and conflicting evidence on the practical impact of this kind of surgery on both overall survival and breast-cancer-specific survival [24,25,26,27,28,29,30,31]. As a consequence, based on the evidence of likely contralateral breast cancer risk reduction and an overall survival increase, the current National Comprehensive Cancer Network (NCCN) guidelines recommend that CRRM should be offered as a choice to patients with a BRCA mutation already diagnosed with breast cancer according to formal consensus [32]. In addition, contralateral prophylactic breast irradiation in patients who oppose CRRM could also be offered for contralateral breast cancer risk reduction, with advantages such as being non-disfiguring and less invasive, but with an uncertain survival benefit [33,34].

### 3.2. Prophylactic Ovarian Surgery

Risk-reducing salpingo-oophorectomy (RRSO) consists of the complete removal of both ovaries and fallopian tubes up to their outlets at the level of the uterine cones. The importance of fallopian tube removal lies in their proven central role in cancer pathogenesis. In fact, there is emerging evidence that ovarian cancer among BRCA mutation carriers predominantly arises from the fallopian tube epithelium and spreads to the ovary secondarily [35,36,37]. The rationale to propose RRBSO to BRCA mutation carriers is that, to date, no cost-effective screening method exists to promptly detect ovarian cancer at an early stage [38,39,40]. It therefore has a very poor prognosis with less than a 50% 5-year survival rate [38,41]. Thereafter, while BRRM is commonly offered as an option by the current international guidelines, RRSO is universally recommended [42]. 

The cumulative lifetime risk of ovarian cancer in the general female population is estimated at 1.3%, increasing up to 36–53% and 10–25% by the age 70 in BRCA1 and BRCA2 mutation carriers, respectively [8,43,44,45], but it is basically unknown for the remaining majority of patients diagnosed with breast cancer who do not carry any pathogenetic BRCA variant. 

In contrast to the general population, ovarian cancer in BRCA mutation carriers is diagnosed at an earlier median age (54 and 59.5 years for the BRCA1 and BRCA2 mutations, respectively, vs. 63 years for wild-type BRCA) [8]. The most frequent histotypes are high-grade serous carcinoma (67%) and endometrioid carcinoma (12%) [46]. 

A prophylactic salpingo-oophorectomy reduces the risk of ovarian cancer by more than 96%, reduces the risk of breast cancer by 72% in patients with the BRCA2 mutation, reduces the risk of breast cancer by 39% in patients with the BRCA1 mutation, and also reduces the overall cancer-specific mortality [47,48,49]. Furthermore, the prevalence of occult cancers found after this type of procedure is about 2–10%, and occult early carcinomas were more frequently found localized to the distal fallopian tube [50,51,52,53].

The timing of RRSO is not universally established; however, there is agreement that it should not be performed before the age of 35 or before completing childbearing [20] (Figure 3). Delaying this type of intervention until physiological menopause should be considered in selected cases if patients are at high risk of cardiovascular events, bone mass density loss, or any other side effect of early menopause. Moreover, a recent multicentric preference study offered BRCA mutation carriers the choice between the standard RRSO or the novel strategy of a risk-reducing salpingectomy with a delayed oophorectomy [54,55,56]. Delayed oophorectomy also may currently be taken into consideration in the case of other gene mutations predisposing patients to gynecological malignancies, such as MLH1, MSH6, and PMS2 in Lynch syndrome [57,58] and PALB2, whose cancer risk range estimates overlap with BRCA [59]. 

This option, however, must be regarded as experimental. It is still unclear how effectively it reduces cancer risk. The authors demonstrated in a simulation study that RRSO is the most effective option for cancer risk reduction [60]. Simultaneously, a risk-reducing salpingectomy combined with a delayed oophorectomy was an excellent option for balancing risk reduction and quality of life [60]. One of the ongoing studies recently published the effects of salpingectomy vs. RRSO on quality of life and demonstrated that QoL was better in the salpingectomy group than in the RRSO group [61]. However, it is known that new ovarian neoplasms can develop not only after salpingectomy but also after RRSO [62,63]. Looking at the ongoing studies, the efficacy analysis in risk reduction is a long-term balance, with the end dates of the ongoing studies planned beyond 2030 [62,63]. Meanwhile, only limited data on cancer risk reduction in this group of high-risk women are currently available [62,63]. Before considering this approach outside of a study protocol, more information is needed. 

### 3.3. Prophylactic Uterine Surgery

Among breast cancer survivors (not screened for germline mutations), increased risks of developing uterine or ovarian cancer higher than 150% and 40%, respectively, have been observed compared to the general population [64]. A recent review found that BRCA mutations were identified in 4.3% of women with endometrial cancer [65]. Moreover, a genomic mutation study that evaluated BRCA-associated mutational and transcriptomic profiles demonstrated the prognostic role of BRCA pathogenetic variants in patients affected by endometrial carcinoma, reporting lower levels of immune cell infiltration, higher expression of immunosuppressive checkpoint molecules, and worse prognoses in the presence of BRCA-associated mutations compared with wild-type BRCA [66]. 

The potential link between BRCA1/BRCA2 mutations and endometrial neoplasia has recently been investigated [67,68,69,70,71]. A cohort study that included 2609 women found no increase vs. the expected risk in the general population [68], but a subsequent meta-analysis found that the BRCA1/BRCA2 mutations increased the risk of any uterine cancers by 2.2 times and increased the risk of serous endometrial neoplasms by about 18 times [69]. Similarly, in a large cohort of BRCA1/2-mutated families, the 5980 individuals carrying the BRCA1/2 mutations had 2.37- and 8.8-fold increased risks of any and serous endometrial carcinoma, respectively, in comparison to the 8541 women not carrying a BRCA mutation [67]. When compared to the risk expected in the general population, women carrying a BRCA 1/2 mutation had 2.83- and 9.77-fold increased risks of any and serous endometrial carcinoma, respectively [67]. The risk was more evident in BRCA1- than BRCA2-mutated individuals [67,69].

On these bases, prophylactic hysterectomy has been proposed as a possible risk-reduction surgery to be discussed in the counseling of BRCA1/2 mutation carriers undergoing RRSO [69,70,72]. Current evidence has also been published about the potential role of risk-reducing hysterectomy in patients carrying other predisposing genetic mutations, such as female carriers of PALB2 variants [73]. A prophylactic hysterectomy consists of completely removing the uterus and the cervix. It can be performed through different access modalities, including laparotomic, laparoscopic, or transvaginally, based on individual indications, and it can be combined with RRSO. Prophylactic hysterectomy carries disadvantages and advantages that should be individually discussed with the woman.

Women considering a hysterectomy are concerned about the effects on their sexual functioning [74]. Indeed, two studies discovered that the most common preoperative anxiety concerned post-hysterectomy sexual functioning [75,76]. Although some evidence suggests that hysterectomy has an unfavorable impact on sexual functioning, other evidence indicates the opposite. Some authors reported declines from 13% to 37% in sexual activities following hysterectomy [74,77,78], and these effects were attributed to a reduction in vaginal length [79] or the removal of nerve endings of the uterovaginal plexus, hampering internal orgasm [80]. Indeed, the same studies reporting deterioration in women’s sex lives after hysterectomy also reported that 16% to 47% of women had no change in their sex lives after hysterectomy and 34% to 70% of women had improvements [74,77,78]. Improvements were sometimes attributed to relief from dyspareunia caused by an excised pelvic pathology [81]. Helstrom and colleagues discovered a link between prehysterectomy dysmenorrhea and post-hysterectomy sexuality and concluded that dysmenorrhea relief leads to improved sexual functioning [82]. According to Richards, patients with increased libido after a hysterectomy expressed relief from pregnancy anxiety [83]. Rhodes and colleagues discovered that sexual functioning improved after hysterectomy in a study of 1299 women [74]. Furthermore, the frequency of sexual activity increased, while problems with sexual functioning decreased [74]. Thus, accordingly to the literature, there is no clear indication that a hysterectomy may either worsen or improve sexual function. On the other hand, alterations in sexual function can be related to RRSO, menopausal hormone withdrawal, and its accompanying changes, which are associated with a progressive worsening of sexual function [84] and can be mitigated by hormonal therapy [85,86,87].

Another issue is the consequence of hysterectomy on pelvic statics. In a nationwide study, Husby and colleagues recently examined 9535 hysterectomies and 47370 controls [88]. This study found that hysterectomy, regardless of parity, is a risk factor for pelvic organ prolapse and that this increased risk is also found in subtotal hysterectomies [88]. The magnitude of the risk for vaginal hysterectomy or laparoscopic-assisted vaginal hysterectomy is greater than for total or subtotal abdominal hysterectomy [88]. All these studies did not include the pelvis statics’ condition before surgery and should be biased by pre-existing pelvic floor defects. However, it should be considered that RRSO-induced menopause is a significant risk factor for prolapse and pelvic floor dysfunction [89,90]. Local or systemic hormones can alleviate symptoms associated with pelvic floor dysfunction caused by surgical menopause [91], and non-invasive treatments such as pelvic rehabilitation can be efficacious [92].

Prophylactic hysterectomy may increase the number of surgical complications. In a large cohort study comprising 78,577 hysterectomies, the total complication prevalence was 10.5% [93]. The hemorrhagic and accidental puncture or laceration of structures accounted for 2.93% of cases [93]. In a series of isolated adnexal surgery, the total prevalence of intraoperative complications was 1.7% [94]. In a nationwide Canadian study to assess urinary tract injuries during benign gynecological surgery, hysterectomy was associated with a significantly higher prevalence of injuries (0.74%, 95% CI 0.67–0.80%) than adnexal surgery (0.10%, 95% CI 0.08–0.12%) [95]. Although the absolute values are low in both groups, the difference was statistically significant, and the urinary tract injuries were associated with a substantial increase in the litigation rate [95]. When counseling women for risk-reduction surgery, these issues should be considered and well-balanced in decision making. However, all those studies were performed in women with uterine pathologies and pelvic floor defects. At the moment, there are no data on the risk associated with a hysterectomy performed only for prophylactic reasons.

Considering the perioperative mortality of this kind of abdominal surgery, which indeed overcomes that of prophylactic breast demolition, some authors investigated the effect of hysterectomy plus RRSO on breast cancer survivors using an Australian population-based data linkage study of 21,067 women diagnosed with primary breast cancer between 1997 and 2008, 1426 of which underwent risk-reducing surgery (13% of premenopausal women and 3% of postmenopausal women) [96]. They ascertained that hysterectomy plus RRSO significantly reduced the risk of overall mortality (HR 0.69; 95% CI 0.53–0.89; *p* < 0.005), which was halved among premenopausal women (HR 0.45; 95% CI 0.25–0.79; *p* < 0.006) and was especially driven by a reduction in breast-cancer-specific mortality (HR 0.43; 95% CI 0.24–0.79; *p* < 0.006). The same differences were confirmed in an independent Australian cohort [97]. A recent systematic review did not provide strong evidence in favor of performing a routine hysterectomy at the time of the risk-reducing surgery [65], but these conclusions should be revised after the publication of new evidence [67,69,70,72]. Undoubtedly, prophylactic hysterectomy, due to its costs and potential complications (bleeding, infection, organ lesions, and vaginal cuff dehiscence), should be individually balanced with the potential increased risk of uterine cancer in this population.

### 3.4. Follow-Up after Risk-Reducing Surgery

Considering the fact that prophylactic strategies succeed but do not completely eliminate the risk of developing hereditary neoplasms, the role of follow-up in this group of high-risk patients, even after any prophylactic interventions, is not negligible.

In a survey of surveillance schemes after risk-reducing surgery from 22 centers across 16 countries and 4 continents, most participating centers agreed that BRCA mutation carriers should not be subjected to active surveillance following risk-reducing surgery [98]. Most centers offered annual clinical breast exams. In contrast, four centers provided annual MRIs, especially for patients with significant residual breast tissue following BRRM. Only four centers provided post-RRSO gynecological surveillance [98]. Moreover, for the gynecological follow-up, there is no agreement [99]. Further evidence is required to improve management after risk-reducing surgery [98].

After prophylactic breast surgery, we strongly advise continuing imaging follow-up by undergoing regular bilateral breast ultrasounds, alternating with MRI if indicated by the breast radiologist [100].

We recommend an annual pelvic ultrasound examination and serum Ca125 testing after RRSO. Cervical cancer screening should be continued in the case of uterus conservation. Furthermore, bone density testing and careful consideration of postmenopausal symptoms are required. Hormone therapy for menopause symptoms is covered in more detail below.

### 3.5. Economic Impact

Preventing the development of new primaries among patients with a recognized predisposition to breast and gynecologic malignancies is of obvious importance in containing the costs related to treating such pathologies, especially at an advanced stage. However, the variability in the type of prevention that can be chosen based on the economic possibilities of the patient and her country of origin is not equally apparent.

For instance, prophylactic surgery can be more convenient than a long-term, frequent, clinical–instrumental follow-up, especially considering the average life expectancy in some low–medium-income countries. In addition, it can be more effortless by considering the low compliance in screening programs, which affects some populations for cultural or geographic reasons. As aforementioned, risk-reducing surgery does not entirely avoid cancer risk; follow-up is nonetheless suggested. However, there is still a need for explicit agreement on the best follow-up after risk-reducing surgery, and new studies are required [98]. The economic balance is dependent on this point.

Furthermore, a recent value of information analysis showed high decision uncertainty associated with the uptake rates of risk-reducing interventions, suggesting that in the future this uptake rate should be given more attention in the conceptualization of health economic modeling studies [101].

### 3.6. Ethical Considerations

In the era of patient-centeredness, it is certainly not possible to ignore the informed and conscious choice of the patient herself. Therefore, it is very important to give her the information in a precise way, starting from the fact that risk-reducing surgery, as the term states, reduces but does not entirely nullify the risk of developing a specific type of tumor due to ectopic tissue, as can happen, for instance, in the case of breast, endometrium, and ovary tissues. Moreover, when opting for risk-reducing interventions, the patient should be led to weigh the benefit of cancer risk reduction against the potential negative consequences of these procedures, including fertility loss, premature menopause, and psychological and physical suffering, in order to eventually opt either for delaying preventive surgery or for carrying on exclusively with intensive surveillance instead [102,103].

Furthermore, the aspects relating to surgical complications and, in the case of breast reconstruction, short- and long-term aesthetic complications cannot be neglected. They should be extensively discussed with the patient to prevent false expectations. Then, the patient must be given the necessary time to metabolize the various options and choose the path she wishes to undertake with complete autonomy, knowing her own risks in case she decides to postpone any preventive intervention.

In general, the literature shows significant variability in risk-reducing surgery uptake among BRCA mutation carriers around the world. These surgeries are widely accepted among women and physicians in Western countries, while they are less accepted in low–middle-income countries, thus reflecting remarkable cultural heterogeneity across countries [101]. Recent evidence also indicates an improved acceptance trend of preventive surgeries over time, probably due to the progressive improvement in genetic counseling protocols and cross-center knowledge transfer [104]. For BRRM, the highest uptake rate is reported in the United States (50%), likely favored by the so-called Angelina effect [105], and the lowest rate is reported in Poland (4.5%) [106]. For RRSO, the highest uptake rate is reported in France (83%), and the lowest rate is reported in China (37%) and in low–middle-income countries [106]. These heterogeneous prevalences emerge despite the current standardized recommendations for this last procedure among BRCA mutation carriers [104,106,107,108,109].

A recent Malaysian study concluded that RRSO decision making involves negotiating the likelihood of developing cancer with the societal priorities of being a woman, mother, and wife [110]. In particular, many interviewed women reported hesitancy toward RRSO and fears about its postoperative, physical, and emotional impacts on their motherhood responsibilities. However, they felt somehow obliged to undergo prophylactic surgery for the sake of their children. Moreover, women’s decisions about choosing this option evolved as their priorities changed at different stages of life. Another Korean study comparing the uptake of BRCA testing, RRSO, and BRRM among the general public, cancer patients, and healthcare professionals highlighted the requirement to develop targeted educational materials and counseling strategies to facilitate informed decision making [111].

### 3.7. Alternative Non-Surgical Prophylactic Options

Historically, risk-reducing ovarian ablation, in addition to the surgical removal of the ovaries, included radiation treatment, GnRH analogs, and chemotherapy. However, ovarian ablation using radiation is less reliable than surgery, and it is associated with increased morbidity and the risk of secondary cancer [112]. Although pharmacological ablation is widely believed to be at least as effective as surgical ablation, surgery is primarily used in a risk-reduction setting for women with an increased risk of both breast and ovarian cancer due to BRCA1 or BRCA2 mutations [112,113,114]. Multiple studies have confirmed this effect, and a meta-analysis revealed a 50% reduction in breast cancer risk [115]. Although there is evidence of GnRH agonists’ potential efficacy in this setting, no large-scale preventative trials have been conducted [116]. One hypothesis was to use GnRH agonists in combination with low-dose estrogen, progesterone, and testosterone to counteract the detrimental effects of ovarian suppression without entirely eliminating risk reduction. A small pilot study of such a protocol in BRCA1 gene mutation carriers found a substantial decrease in mammographic density (breast cancer risk surrogate) with no adverse effects on quality of life or bone mineral density [117]. However, more data are required before this treatment may be developed as an opportunity for women who are not yet ready to undergo surgical risk reduction.

Systemic chemopreventive schemes use drugs that inhibit sex hormones in different ways. During the fertile age, tamoxifen is used in high-risk women over the age of 35, except in cases where there is a known family history of thromboembolic events and a family or personal history of endometrial cancer. Tamoxifen, a selective estrogen receptor modulator (SERM), may be an option for these very young women, but it is associated with significant side effects (e.g., hot flashes, endometrial cancer, venous thromboembolic disease, depression, diminished sexual functioning, etc.), and there are no trial data on its efficacy in women under the age of 35 [32,118]. Chemoprevention using tamoxifen was demonstrated to reduce contralateral breast cancer risk by 62% in BRCA1/2 mutation carriers [32]. In addition, it reduced cancer-related mortality by about 18% [119]. Unfortunately, BRCA pathogenetic variants, especially those of BRCA1, may predispose their carriers to more frequently developing triple-negative malignancies and thus are commonly unresponsive to antihormonal prophylactic schemes. Moreover, data from the literature show that antihormonal chemoprophylaxis use in BRCA mutation carriers remains low because of its evident side effects on the quality of life [120].

Aromatase inhibitors are restricted to postmenopausal women because constitutive estrogen synthesis occurs in peripheral body fat during this period and aromatase inhibitors can block this [32,121]. Meanwhile, during the fertile age, other regulatory mechanisms are involved in estrogen and progesterone synthesis that can avoid the effects of aromatase inhibitors [121]. Furthermore, raloxifene’s effectiveness in premenopausal women is unknown because it has only been studied in postmenopausal women [32]. After menopause, on the other hand, tamoxifen is considered in previously hysterectomized women. Raloxifene is considered in those with the uterus still in place, or aromatase inhibitors are considered in the case of high thromboembolic risk [32]. In patients taking aromatase inhibitors, bone mass monitoring is necessary, given their increased risk of demineralization and the subjective point of view of patients with diffuse pain.

The chemopreventive treatment duration should be based on the available evidence. Currently, chemopreventive efficacy data are based on a 5-year daily treatment [122,123,124,125,126]. Recently, it was found that a 5-year chemopreventive treatment maintains a long-term beneficial effect at a 10-year follow-up [127].

In addition to the chemoprevention of the breast neoplasm, it should be highlighted how the use of low-dose oral contraceptives involves a significant reduction in the risk of ovarian neoplasm without increasing the risk of breast neoplasm [20]. In BRCA1 mutation carriers, oral contraception lowered the risk of ovarian cancer by 45–50%, and in BRCA2 mutation carriers, it decreased the risk by 60% [20].

Follow-up plays a crucial role in all BRCA mutation carriers who decide to conserve their breast and gynecological apparatuses. For these patients, international guidelines call for an annual breast screening MRI starting at age 25, whereas the age of onset for yearly mammograms differs between 30 and 40 or possibly 10 years earlier than the first case in the family [42,128]. Individuals identified with variants of unknown significance (VUS) should be counseled based on their personal and family histories, irrespective of the variant [129].

### 3.8. Hormone Therapy for Menopause Symptoms

In a recent population-based study, Öfverholm and coworkers analyzed mortality in a cohort of women treated with risk-reducing surgery (BRRM and RRSO) [130]. The mean age at RRSO was 43.4 years (range: 28.2–79.7) [130]. Although they found a reduction in breast and ovarian cancer incidence and mortality after BRRM and RRSO, they also found significantly increased overall mortality rates compared to general population of age-matched women [130]. In general, surgical menopause under 45 years is associated with the worst menopausal symptoms and an increase in cardiovascular mortality [85,86,87,131,132,133].

Hormone therapy (HT) can be given to counteract the symptoms of early menopause, and it is also known to reduce mortality due to cardiovascular events when given at an early age or immediately after menopause [85,86,87,134]. HT alleviates menopausal symptoms, sexual dysfunction, bone loss, and the risk of fractures at any skeleton site [85,86,87]. It lowers the risk of cardiovascular disease when started within 10 years of menopause or before 60 years [85,86,87]. Furthermore, Rocca and colleagues discovered that women who had a bilateral prophylactic oophorectomy before age 45 were more likely to die from non-cancer causes [133]. Meanwhile, no increase in mortality was observed in women who received estrogen therapy until the age of 45 [133]. These benefits, along with the addition or reduction in cognitive impairment, are more pronounced in women experiencing early or premature menopause, including those caused by ovariectomy [85,86,87,132]. Two systematic reviews concluded that HT does not counteract the risk reduction associated with surgery [134,135].

So far, the cost–benefit analysis for prophylactic uterine surgery has focused primarily on the potential reduction in risk associated with endometrial cancer, particularly the serous type. In our opinion, other elements must be taken into consideration. For example, suppose we remove the ovaries and the uterus in the same surgical session. In that case, it involves submitting the patient to HT only with estrogen without progestins. A recent review including observational studies found that women who received estrogen alone tended to have a lower risk of breast cancer than women who received estrogen plus progesterone [135]. In an analysis of two randomized clinical trials considering 27,347 postmenopausal women, estrogen-only HT was linked to a lower risk of breast cancer compared to combined estrogen and progestin, which is required when the uterus is left in place [136]. Indeed, epidemiological evidence also indicates that certain synthetic progestins used to protect the endometrium may increase breast cancer risk when combined with estrogens [137,138]. Given the possible link between progestin use in HT and breast cancer risk, if a hysterectomy has been performed, HT should be based solely on estrogens (systemic or topical) and should not include progestins. This is particularly important if we think that residual glandular breast tissue is reported in up to 100% of mastectomy patients, as reported in a recent systematic review [12]. Residual breast tissue can be found in the remaining chest wall (e.g., in the skin flaps or beneath the nipple–areolar complex) [12]. This reality puts these women at risk of developing breast cancer, despite this risk being reduced because the gland mass is minimal [139]. In a study published in 2019, Papassotiropoulos and coworkers found residual breast tissue in 51.3% of mastectomies, and the residual breast tissue percentage per breast was 7.1% on average [13]. Potential advantages of overcoming the need for a hysterectomy may derive from substituting progestins with bazedoxifene, a selective estrogen receptor modulator that is able to protect the endometrium from estrogen stimulation and exert possible benefits for the breasts. However, keeping the uterus in place would force women to only one type of HT combination (conjugated equine estrogen and bazedoxifene), not allowing individualization of the treatment in terms of doses, molecules, and routes of administration [86]. Another novelty is the introduction of estetrol, a native estrogen with selective tissue activity that has a limited impact on breast tissue [140]. As a result, it may play a role in HT in this group of women. Nonetheless, it is too early to draw definitive conclusions because it is a relatively new drug in this area.

## 4. Conclusions

### 4.1. The Breast Surgeon’s Perspective

The literature shows several shreds of evidence related to the efficacy of a combined risk-reducing surgical strategy. However, one cannot ignore the risks of abdominal–pelvic surgery compared to breast surgery alone and the systemic risks dictated by the interruption of ovarian function in premenopausal women. Furthermore, excluding the case of healthy BRCA mutation carriers, in our opinion, the complications of risk-reducing gynecological surgery would not justify any delay in initiating adjuvant treatments for breast cancer when appropriate. Therefore, although reducing the number of interventions may seem advantageous at first, the timing of the various types of interventions should probably be better defined and undoubtedly discussed within a multidisciplinary setting, also taking into account the individual cardiovascular risk factors and the women’s personal desires as well as considering the possibility of salpingectomy with delayed ovariectomy, which has recently been explored.

For prophylactic mastectomy, it is essential to point out that this procedure serves to contain the risk of developing breast cancer but does not entirely cancel it. Therefore, in this case, it is also good to consider that even this type of intervention is not completely free of complications, in addition to the fact that in breast cancer survivors the prognosis is certainly guided by the carcinoma that is already diagnosed and not by any carcinomas not yet in place.

Finally, each patient should undergo an individualized risk assessment, possibly within a multidisciplinary setting. The most appropriate risk-reduction option should be chosen considering the patient’s goals, risk profile, and risk tolerance. In addition, we believe that even the VUS, considered negative for the moment, deserves a multidisciplinary discussion with peculiar decisions that must be taken on a case-by-case basis, such as in patients with a marked familial history or carcinomas at a very young age.

### 4.2. The Gynecologist’s Perspective

Oral hormonal contraception should not be negated in BRCA1/BRCA2 mutation carriers not yet eligible for risk-reducing surgery because it significantly reduces the risk of developing ovarian cancer. Although prophylactic surgery reduces the risk of mammary neoplasm in BRCA1/BRCA2 mutation carriers, it has drawbacks related to early menopause. Prophylactic hysterectomy, in addition to lowering the risk of developing a serous endometrial neoplasm, allows for the use of estrogen-only hormonal therapy to alleviate the complications of early menopause.

A multidisciplinary team comprising a breast specialist, a gynecological oncologist, and a gynecological endocrinologist must carefully inform the woman who chooses this path of the wide range of implications ranging from cancer risk reduction to hormonal therapies.

### 4.3. Multidisciplinary Approach to Risk Reduction

We believe that a “one-size-fits-all” strategy is impossible to achieve; the timing of various types of interventions should be defined and discussed in a multidisciplinary setting, taking individual risk factors and personal desires into account, and the team should inform the women about all possible implications. We believe separating breast surgeries from ovary, fallopian tube, and uterus surgeries is advantageous. If the annexes are removed, there is an advantage to removing the uterus as well, at the expense of an increase in surgical complications. Each patient should have an individualized risk assessment, and the best risk-reduction option should be chosen based on their goals, risk profile, and tolerance.

## Figures and Tables

**Table 1:** Queries used to retrieve the literature items.

Database	Query	Date of Retrieval	Number of Items
PubMed/Medline	((BRCA OR “genetic carrier” OR “genetic predisposition” OR “germ-line mutation” OR hereditary) AND (breast)) AND ((mastectomy OR salpingo-oophorectomy OR hysterectomy OR surgery) AND (risk-reducing OR “risk reducing” OR risk reduction OR prophylactic)) AND ((breast OR mammary OR endometrial OR endometrium OR ovary OR ovaries OR ovarian OR uterine OR uterus OR fallopian tube* OR genital) AND (cancer* OR neoplasm* OR carcinoma*))	06 August 2022	1519
Scopus	((BRCA OR “genetic carrier” OR “genetic predisposition” OR “germ-line mutation” OR hereditary) AND (breast)) AND ((mastectomy OR salpingo-oophorectomy OR hysterectomy OR surgery) AND (risk-reducing OR “risk reducing” OR risk reduction OR prophylactic)) AND ((breast OR mammary OR endometrial OR endometrium OR ovary OR ovaries OR ovarian OR uterine OR uterus OR fallopian tube* OR genital) AND (cancer* OR neoplasm* OR carcinoma*))	06 August 2022	2501
EMBASE	AB,TI((BRCA OR “genetic carrier” OR “genetic predisposition” OR “germ-line mutation” OR hereditary) AND (breast)) AND AB,TI((mastectomy OR salpingo-oophorectomy OR hysterectomy OR surgery) AND AB,TI(risk-reducing OR “risk reducing” OR risk reduction OR prophylactic)) AND AB,TI((breast OR mammary OR endometrial OR endometrium OR ovary OR ovaries OR ovarian OR uterine OR uterus OR fallopian tube* OR genital) AND (cancer* OR neoplasm* OR carcinoma*))	06 August 2022	1618

## Data Availability

All data were extracted from previously published studies or public databases; thus, they are publicly available.
